# Enhanced Facility-Based Hypertension Screening: A Quality Improvement Initiative at Primary Health Care Facilities in Ethiopia

**DOI:** 10.5334/gh.1584

**Published:** 2026-09-23

**Authors:** Workneh Demissie, Henok G. Kebede, Naod Wendrad, Bishal Belbase, Assefa Gire, Wondwosen Berhe, Selamawit Ayele, Bolanle F. Banigbe, Girma A. Dessie, Reena Gupta

**Affiliations:** 1 Resolve to Save Lives, Country Office, Addis Ababa, Ethiopia; 2 Resolve to Save Lives, VA, USA; 3 Oromia Health Bureau, Ethiopia; 4 Addis Ababa City Administration Health Bureau, Ethiopia; 5 Ministry of Health Ethiopia, Ethiopia; 6 University of California San Francisco, San Francisco, CA, USA

**Keywords:** Hypertension Screening, Quality Improvement, Primary Health Care, Ethiopia

## Abstract

Less than half of people with hypertension in Ethiopia are diagnosed, and only one-third of adults visiting primary care facilities have their blood pressure (BP) screened. We implemented a quality improvement (QI) initiative across six facilities in Ethiopia to increase facility-based BP screening. Process mapping identified barriers, and interventions were tested in iterative QI cycles: establishing BP screening desks at key service points, ensuring the availability of validated BP devices, training staff in accurate measurement, standardizing documentation, and reviewing screening indicators in performance meetings. Between April 2024 and February 2025, monthly BP screening of adults without a hypertension diagnosis increased from 41.0% of 13,707 eligible adults to 79.6% of 14,270 eligible adults. Monthly hypertension program enrollments rose from 40 in April 2024 to 125 in February 2025. Strengthening primary health care systems to systematically measure BP for all adults visiting health facilities can significantly improve hypertension detection and treatment.

## Background

Hypertension is the world’s leading risk factor for preventable death (1,2). Over half of adults with hypertension in Ethiopia remain undiagnosed. Of the estimated 11 million people in Ethiopia living with high blood pressure (BP) (16% of the adult population), only 40% are aware and less than 2% have their BP under control (3). Early diagnosis through screening and prompt management of hypertension are crucial to reducing the burden of disabling heart attacks, strokes, and premature death from cardiovascular disease (CVD) in Ethiopia (2).

The Ethiopia Federal Ministry of Health (FMoH), in partnership with the global non-profit organization Resolve to Save Lives (RTSL), implemented the Ethiopian Hypertension Control Initiative (EHCI), a World Health Organization (WHO) technical Package of HEARTS (H-Healthy Lifestyle counselling, E-Evidence-based treatment protocols, A-Access to essential medicines and technology, R-Risk-based CVD management, T-Team-based care, S-Systems for monitoring) module-based program to enhance hypertension care in 2019 (4,5). The HEARTS technical package advocates for simple treatment protocols, reliable supplies of medicines and validated BP measurement devices, team-based care, patient-centered care, and a robust health information system. Facility-based BP screening was implemented as a core component when the EHCI program was launched, aiming to strengthen hypertension screening within the existing primary healthcare system.

Facility-based BP screening is the practice of measuring the BP of every adult without a known hypertension diagnosis at every health visit. Unlike more resource-intensive community-based screening, facility-based screening can be integrated into usual clinic workflows and is a cost-effective strategy that improves early hypertension diagnosis and linkage to treatment (6,7,8). However, without consistent facility BP screening, far too often adults who are unaware that they have hypertension visit health facilities without being screened or diagnosed (9,10,11). There is a paucity of literature on effective strategies to improve the coverage of facility BP screening rates in primary health facilities in low- and middle-income countries (LMICs).

An assessment of the EHCI program in December 2023 in Addis Ababa and the Oromia region revealed that despite having a dedicated BP screening protocol, only 32% of adults 18 years or older without a hypertension diagnosis and visiting primary health care facilities were screened for high BP during their routine health facility visit. The EHCI was far from reaching the program’s target of 80% BP screening, representing significant missed opportunities for hypertension detection. To improve facility-based BP screening in the EHCI program, we implemented a structured quality improvement (QI) initiative in six primary health care facilities in the Addis and Oromia regions of Ethiopia.

## Method

### Design

We conducted a QI initiative among six primary health facilities, three in Addis Ababa city administration and three in Oromia region, participating in the EHCI program between April 2024 and February 2025. We used the Model for Improvement (MFI) framework aligned with the Ethiopian National Healthcare Quality and Safety Strategy (2021–2025) (12,13,14). The framework includes: (1) setting aims, (2) establishing measures, (3) selecting changes through process mapping and root cause analysis, and (4) testing changes through iterative Plan-Do-Study-Act (PDSA) cycles of QI. The RTSL program managers engaged health facility leadership and local health office authorities to create awareness, ensure alignment, and drive action. QI team members included national and sub-national hypertension program leaders, government officials, and facility staff from each of the six facilities.

### Setting aims

We aimed to improve facility-based BP screening in the six primary health facilities from the baseline of 41% in April 2024 to 80% by February 2025.

### Establishing measures

We included all adults aged 18 years and older who attended outpatient departments of selected facilities. We excluded those previously diagnosed with hypertension and women attending antenatal clinics since BP measurement is a routine part of care in these clinics.

We calculated monthly facility BP screening by dividing the number of adult individuals (age 18 and older) who were screened for hypertension during the month by the number of eligible adult individuals who visited the health facility during the month. We measured the number of new individuals diagnosed and enrolled in the hypertension program. We received aggregate monthly de-identified data on the number of adults screened and the number of adults who visited the primary care facilities from District Health System Software 2 (DHIS2) and aggregate monthly de-identified data of hypertension patients enrolled in care from Simple mobile application (15).

### Selecting changes: process flow mapping and root cause analysis

**Supplemental Figure 1:**
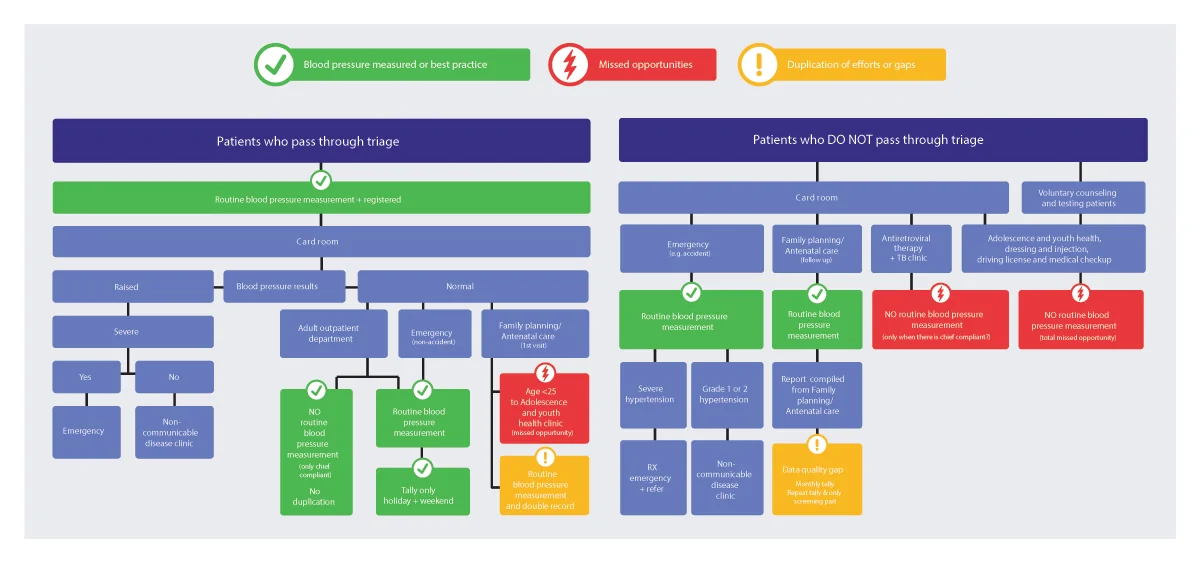
Patient flow mapping exercise: Facility A.

Though the facilities already had a BP screening protocol to measure BP for all patients age 18 and older at existing triage and BP screening stations, many patients were not being routinely screened. To understand patient care flows and identify gaps in existing screening processes and linkage to diagnosis, team members mapped patient and clinical workflows to identify where missed opportunities for hypertension screening and referral were occurring (Supplemental Figure 1).

The patient flow mapping exercise at each facility revealed several challenges and missed opportunities in existing facility-based BP screening workflows. Specific groups of patients bypassed BP screening stations (most commonly adolescents, voluntary counseling and testing, and clients who came for routine injection, driving license, and medical checkups). Routine BP screening was also not routinely integrated in the Human Immunodeficiency Virus (HIV) and Tuberculosis (TB) clinics. Flow mapping additionally revealed workflow inefficiencies due to unnecessary steps in patient flow and duplication of efforts, inconsistent or missing records due to lack of a standardized recording tool, and poor referral linkage of people identified with raised BP for follow-up and diagnosis confirmation.

### Testing changes

Through a series of rapid PDSA cycles, facility teams tested a series of interventions to address the above screening gaps at each facility to increase BP screening. The QI initiative introduced the following new interventions: (1) reorganizing patient flow to route all eligible adults to have their BP measured at triage or dedicated BP screening corners. Dedicated BP screening corners also accommodating screening of eligible adults who accompany patients; (2) integrating routine BP screening at HIV and TB clinics where routine BP measurement was not consistently performed previously; (3) assigning staff for hypertension screening and providing orientation on proper BP measurement, recording practices, and referral linkage for diagnosis confirmation; (4) standardizing the screening outcomes recording tool; and (5) adding BP screening performance monitoring to the agendas of routine health facility performance monitoring team (PMT) meetings to identify ongoing gaps and solve problems. The initiative leveraged existing facility staff by integrating routine BP screening into their routine responsibilities, without recruiting additional human resources. From these six QI-implementing primary health care facilities, routine hypertension screening program performance data were reviewed, and the monthly trend over time was observed before and after the QI initiative was implemented.

## Results

**Figure 1:**
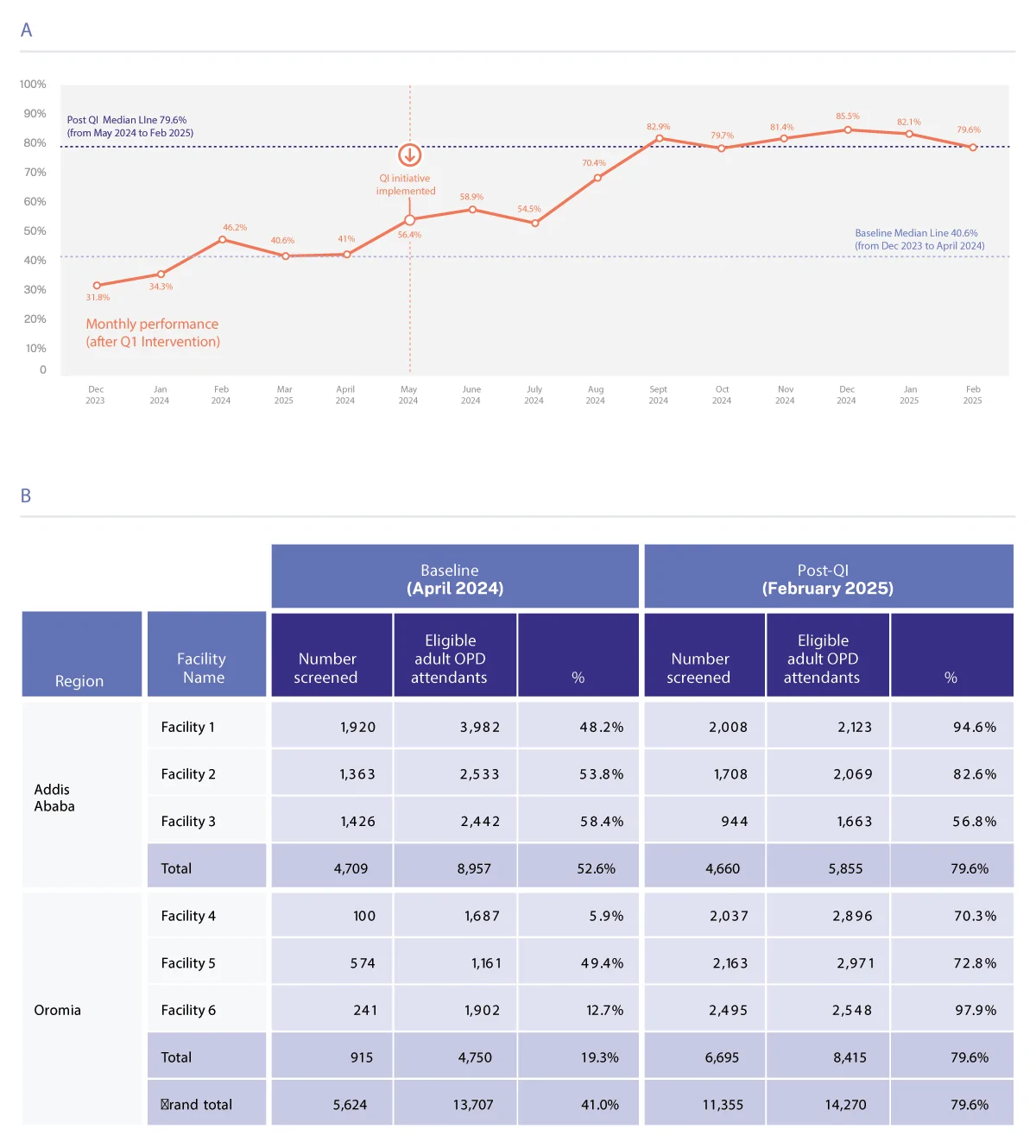
Performance of faciity-based screening before and ten months after quality improvement initiative in six primary health facilities in Ethiopia.

At baseline in April 2024, 5,624 out of 13,707 eligible adults who visited the facility (41.0%) were screened for hypertension. BP screening varied between facilities from 5.9% to 58.4% at baseline (Figure 1). By February 2025, BP screening had increased to 79.6% (11,355 screened out of 14,270 adult individuals who visited the facility). Between facilities, BP screening varied from 56.8% to 97.9% in February 2025 (Figure 1). BP screening increased from 52.6% in April 2024 to 79.6% in February 2025 among the three facilities in Addis Ababa region and increased from 19.3% in April 2024 to 79.6% in February 2025 among the three facilities in Oromia region. Monthly enrollments in the hypertension program among the six facilities increased from 40 people in April 2024 to 125 people in February 2025. Nine hundred seventy cumulative people were diagnosed with hypertension and started treatment from April 2024 to February 2025 in these six facilities.

## Conclusions

Only 41% of Ethiopian adults visiting six primary health care facilities were screened for hypertension at baseline, representing significant missed opportunities for hypertension detection. With a structured QI approach and implementation of specific interventions, facility-based BP screening increased from 41% to 80%.

Numerous studies have shown that universal facility BP screening results in more patients with hypertension being diagnosed, treated, and controlled (6,7,8,9,10). Common barriers cited in these studies explaining low screening coverage are similar to those encountered in our program: limited staff resources, competing priorities for time with other duties, absence of dedicated screening stations, low awareness among patients, and lack of standardized data collection tools.

There are several limitations of this QI initiative. We were unable to assess the individual effect of each component of the QI interventions on BP screening. We did not document the additional time that existing staff spent on additional BP screening or impact on other duties. However, each of the six facilities reported existing staff were able to incorporate additional BP screening and maintain other staff functions. We acknowledge the improvements in BP screening described in these six facilities may not be generalizable to other regions or countries. The EHCI program is scaling these QI interventions across the program to further assess which components drive improvement at scale.

Our initiative demonstrates the value of a structured QI approach, utilizing process mapping to identify and design interventions addressing root causes of low screening. Standardizing patient flow pathways improved staff efficiency, increased hypertension screening coverage, and enhanced linkage of individuals with raised BP to diagnosis and treatment of hypertension. In addition, systematic performance monitoring with data feedback loops in the primary care facilities drove improvement. Integrating facility-based BP screening delivered by existing facility staff into routine primary care supports sustainability. If 80% facility-based BP screening could be scaled nationwide across the country’s 4,000 primary care facilities, 672,000 additional individuals could be detected and enrolled in hypertension care annually in Ethiopia. The EHCI program is now assessing the long-term sustainability of the QI intervention in the original facilities and scaling up this approach to improve facility-based BP screening across the country.

## References

[1] Murray CJL, Aravkin AY, Zheng P, et al. Global burden of 87 risk factors in 204 countries and territories, 1990–2019: a systematic analysis for the Global Burden of Disease Study 2019. The Lancet. 2020;396(10258):1223–1249. DOI: 10.1016/S0140-6736(20)30752-2 PMC7566194 33069327

[2] World Health Organization. Global Report on Hypertension: The Race against a Silent Killer. Available from: https://www.who.int/publications/i/item/9789240081062. Accessed May 1, 2025.

[3] Ethiopian STEPS report on risk factors for non-communicable disease and prevalence of selected NCDs; 2016. Available from: https://www.who.int/publications/m/item/2015-steps-country-report-Ethiopia. Accessed May 1, 2025.

[4] Resolve to Save Lives. Hypertension Control. Resolve to Save Lives. Available from: https://resolvetosavelives.org/cardiovascular-health/hypertension. Accessed May 1, 2025.

[5] World Health Organization. HEARTS: Technical package for cardiovascular disease management in primary health care: Evidence-based treatment protocols. 2018. Available from: https://apps.who.int/iris/bitstream/handle/10665/260421/WHO-NMH-NVI-18.2-eng.pdf?_sequence1/41. Accessed May 1, 2025.

[6] Frieden TR, Garg R, Moran AE, Whelton PK. Improved hypertension care requires measurement and management in health facilities, not mass screening. Lancet. 2025;405(10492):1879–1882. DOI: 10.1016/S0140-6736(25)00561-6 40347970

[7] Meador M, Osheroff JA, Reisler B. Improving identification and diagnosis of hypertensive patients hiding in plain sight (HIPS) in health centers. The Joint Commission Journal on Quality and Patient Safety. 2018;44:117–129. DOI: 10.1016/j.jcjq.2017.09.003 29499808

[8] WHO. Hypertension care in Thailand: best practices and challenges, 2019. Available from: https://www.who.int/publications/i/item/9789290227403. Accessed May 1, 2025.

[9] Wall HK, Hannan JA, Wright JS. Patients with undiagnosed hypertension: hiding in plain sight. JAMA 2014;312:1973–1974. DOI: 10.1001/jama.2014.15388 PMC4596255 25399269

[10] Maurer J, Ramos A. One-year routine opportunistic screening for hypertension in formal medical settings and potential improvements in hypertension awareness among older persons in developing countries: Evidence from the Study on Global Ageing and Adult Health (SAGE). American Journal of Epidemiology. 2015;181(3):180–184. DOI: 10.1093/aje/kwu339 PMC4312429 25550358

[11] Kaviprawin M, Ramalingam A, Varghese A, et al. Missed opportunities for detection of hypertension in public health facilities of 18 districts in India, 2022. BMC Public Health. 2025;25(1):1082. DOI: 10.1186/s12889-025-22284-4 PMC11927288 40119316

[12] How to Improve: Model for Improvement, Institute for Healthcare Improvement. Available from: https://www.ihi.org/resources/how-improve-model-improvement. Accessed May 1, 2025.

[13] Magge H, Kiflie A, Nimako K, et. al. The Ethiopia healthcare quality initiative: design and initial lessons learned, International Journal for Quality in Health Care. 2019;31(10):G180–G186. DOI: 10.1093/intqhc/mzz127 31834384

[14] Ethiopian National Healthcare Quality and Safety Strategy (2021–2025). Available from: https://repository.iphce.org/handle/123456789/1656. Accessed May 1, 2025.

[15] Burka D, Gupta R, Moran AE, et al. Keep it simple: Designing a user-centred digital information system to support chronic disease management in low/middle-income countries. BMJ Health & Care Informatics. 2023;30(1):e100641. DOI: 10.1136/bmjhci-2022-100641 PMC9843217 36639189

